# Spinal cord stimulation modulates supraspinal centers of the descending antinociceptive system in rats with unilateral spinal nerve injury

**DOI:** 10.1186/s12990-015-0039-9

**Published:** 2015-06-24

**Authors:** Toshiharu Tazawa, Yoshinori Kamiya, Ayako Kobayashi, Kensuke Saeki, Masahito Takiguchi, Yusuke Nakahashi, Hironobu Shinbori, Kengo Funakoshi, Takahisa Goto

**Affiliations:** Department of Anesthesiology, Yokohama City University, 3-9 Fukuura, Kanazawa-ku, Yokohama, 236-0004 Japan; Department of Neuroanatomy, Yokohama City University, 3-9 Fukuura, Kanazawa-ku, Yokohama, 236-0004 Japan; Pain Mechanism Research Group, 1-757 Asahimachi-dori, Chuo-ku, Niigata, 951-8510 Japan; Division of Anesthesiology, Niigata University Graduate School of Medical and Dental Sciences, 1-757 Asahimachi-dori, Chuo-ku, Niigata, 951-8510 Japan

**Keywords:** Descending antinociceptive system, Dorsal raphe nucleus, Locus coeruleus, Neuropathic pain, Noradrenergic pathway, Serotonergic pathway, Spinal cord, Spinal cord stimulation

## Abstract

**Background:**

The descending antinociceptive system (DAS) is thought to play crucial roles in the antinociceptive effect of spinal cord stimulation (SCS), especially through its serotonergic pathway. The nucleus raphe magnus (NRM) in the rostral ventromedial medulla is a major source of serotonin [5-hydroxytryptamine (5-HT)] to the DAS, but the role of the dorsal raphe nucleus (DRN) in the ventral periaqueductal gray matter is still unclear. Moreover, the influence of the noradrenergic pathway is largely unknown. In this study, we evaluated the involvement of these serotonergic and noradrenergic pathways in SCS-induced antinociception by behavioral analysis of spinal nerve-ligated (SNL) rats. We also investigated immunohistochemical changes in the DRN and locus coeruleus (LC), regarded as the adrenergic center of the DAS, and expression changes of synthetic enzymes of 5-HT [tryptophan hydroxylase (TPH)] and norepinephrine [dopamine β-hydroxylase (DβH)] in the spinal dorsal horn.

**Results:**

Intrathecally administered methysergide, a 5-HT_1_- and 5-HT_2_-receptor antagonist, and idazoxan, an α_2_-adrenergic receptor antagonist, equally abolished the antinociceptive effect of SCS. The numbers of TPH-positive serotonergic and phosphorylated cyclic AMP response element binding protein (pCREB)-positive neurons and percentage of pCREB-positive serotonergic neurons in the DRN significantly increased after 3-h SCS. Further, the ipsilateral-to-contralateral immunoreactivity ratio of DβH increased in the LC of SNL rats and reached the level seen in naïve rats, even though the number of pCREB-positive neurons in the LC was unchanged by SNL and SCS. Moreover, 3-h SCS did not increase the expression levels of TPH and DβH in the spinal dorsal horn.

**Conclusions:**

The serotonergic and noradrenergic pathways of the DAS are involved in the antinociceptive effect of SCS, but activation of the DRN might primarily be responsible for this effect, and the LC may have a smaller contribution. SCS does not potentiate the synthetic enzymes of 5HT and norepinephrine in the neuropathic spinal cord.

## Background

Spinal cord stimulation (SCS) is widely performed to manage intractable pain. The antinociceptive effect of serotonin (5-HT) in the spinal cord is well documented in animals [1–4]. Intrathecal 5-HT administration facilitates SCS-induced antinociception [5]. Early studies revealed that electrical stimulation of the brain stem, dorsolateral funiculus, and peripheral nerves activates the serotonergic pathway of the descending antinociceptive system (DAS), with 5-HT release in the spinal cord resulting in an antinociceptive response [6, 7]. Moreover, electrical stimulation at the level of the dorsal column nuclei attenuates the flexor reflex and hypersensitivity after peripheral nerve injury [8, 9]. Therefore, the antinociceptive effect of SCS might involve supraspinal neural circuits via activation of the DAS, which is at least partly serotonergic. The nucleus raphe magnus (NRM) in the rostral ventromedial medulla (RVM) is a major serotonergic center in the brain stem, and its stimulation induces GABA release into the spinal dorsal horn [10] and antinociception [11, 12]. However, the role of the dorsal raphe nucleus (DRN), a source of 5-HT in the ventral periaqueductal gray matter (PAG), still needs to be elucidated.

The noradrenergic pathway of the DAS is activated in animal models of neuropathic pain [13, 14], and α_2_-adrenergic agonists have antinociceptive effects in both animals and patients with neuropathic pain [15–20]. In addition, clonidine, an α_2_-adrenergic agonist, potentiates the analgesic effect of SCS after tactile hypersensitivity in animal models of neuropathic pain [13, 21–24]. However, the contribution of the noradrenergic pathway to SCS-induced antinociception is controversial.

It has been believed that 5-HT and noradrenaline in spinal dorsal horn would be synthesized in the supraspinal center of the serotonergic and noradrenergic nuclei respectively, and transported to the spinal cord. On the other hand, 5-HT [tryptophan hydroxylase (TPH)] and norepinephrine [dopamine β-hydroxylase (DβH)] synthetic enzymes are expressed in the spinal cord, so the corresponding neurotransmitters could be synthesized locally. However, we had little information on how spinal 5-HT and norepinephrine production contributes to SCS-induced antinociception, despite several studies suggesting that these neurotransmitters would be produced in the serotonergic and noradrenergic neurons in brain stem nuclei [25, 26]. Moreover, it is still unclear whether spinal or supraspinal modulation is essential for SCS-induced antinociception, despite recent studies showing that segmental effects in the spinal cord are crucial for antinociception by SCS [27, 28].

To answer these questions, we investigated whether the serotonergic and noradrenergic pathways of the DAS are involved in the antinociceptive effect of SCS by behavioral analyses of spinal nerve-ligated (SNL) rats. To identify the essential supraspinal centers, we performed immunohistochemical analyses of tissues containing the DRN and locus coeruleus (LC), regarded as the adrenergic center of the DAS. Finally, to elucidate whether local synthesis of the neurotransmitters is important for SCS-induced antinociception, we evaluated changes in the expression of TPH and DβH in the spinal dorsal horn with or without SCS by western blot analyses.

## Results

### Methysergide and idazoxan attenuated the antinociceptive effect of SCS

**Figure 1:**
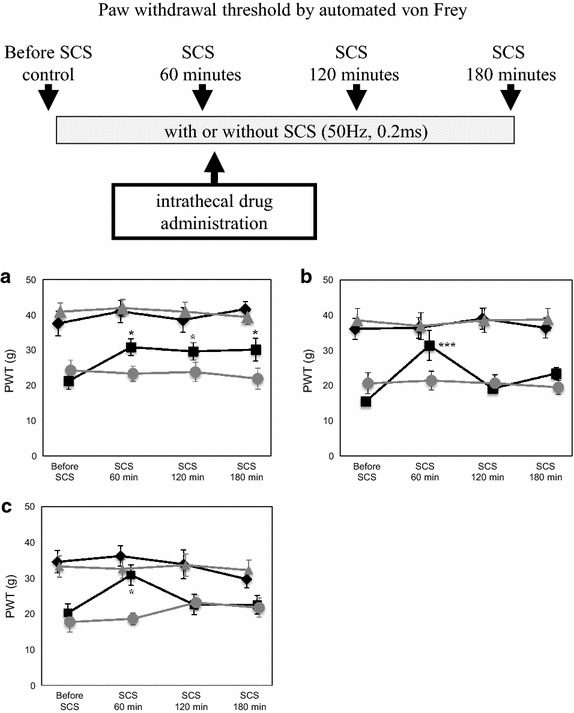
Assessment of mechanical hypersensitivity and antianalgesic effect of 5-HT and α_2_-adrenergic receptor antagonists. **a** Time course of the behavioral experiment. PWT was measured by an automated von Frey device five times on each side. After the baseline measurement, SCS (0.2 ms, 50 Hz, 70–80% of the motor threshold) was initiated and PWT was assessed every hour. Methysergide (5-HT receptor antagonist; 30 μg), idazoxan (α_2_-adrenergic receptor antagonist; 30 μg), or saline was administered as a bolus via intrathecal catheters in freely moving rats immediately after verifying the antinociceptive effect of 1-h SCS. **b** Ipsilateral PWT of SNL rats significantly increased after SCS without influencing contralateral PWT (n = 7). Saline (20 µL) was administered via the intrathecal catheters regardless of SCS. **c** The increase in ipsilateral PWT was completely reversed by intrathecal methysergide administration without influencing contralateral PWT (n = 6). **d** The increase in ipsilateral PWT was completely reversed by intrathecal idazoxan administration without influencing contralateral PWT (n = 6). *Black square* ipsilateral PWT with SCS, *black diamond* contralateral PWT with SCS, *gray circle* ipsilateral PWT without SCS, *gray triangle* contralateral PWT without SCS. Data are mean ± SEM (g); *p < 0.05 and ***p < 0.001 versus before SCS by one-way repeated-measures ANOVA followed by the Dunnett test.

Spinal nerve-ligated (SNL) rats showed ipsilateral mechanical hypersensitivity. SCS significantly reduced the hypersensitivity in about 70% of these rats (baseline paw withdrawal threshold [PWT] = 21.4 ± 2.3 g; after 1-h SCS = 30.9 ± 2.4 g; after 2-h SCS = 29.6 ± 2.4 g; after 3-h SCS = 30.2 ± 3.2 g; *F* [3, 32] = 3.78; *p* = 0.02). It however did not significantly affect contralateral PWT (baseline = 37.5 ± 3.4 g; after 1-h SCS = 40.9 ± 3.1 g; after 2-h SCS = 38.5 ± 3.4 g; after 3-h SCS = 41.5 ± 2.1 g; *F* [3, 32] = 0.43; *p* = 0.73). Significant differences in the antinociceptive effect were noted between the ipsilateral and the contralateral hind paws (baseline PWT difference, *p* = 0.0044; stimulated PWT difference, *p* < 0.001; interaction of side and treatment, *p* = 0.022; n = 9) (Figure 1b).

**Figure 2:**
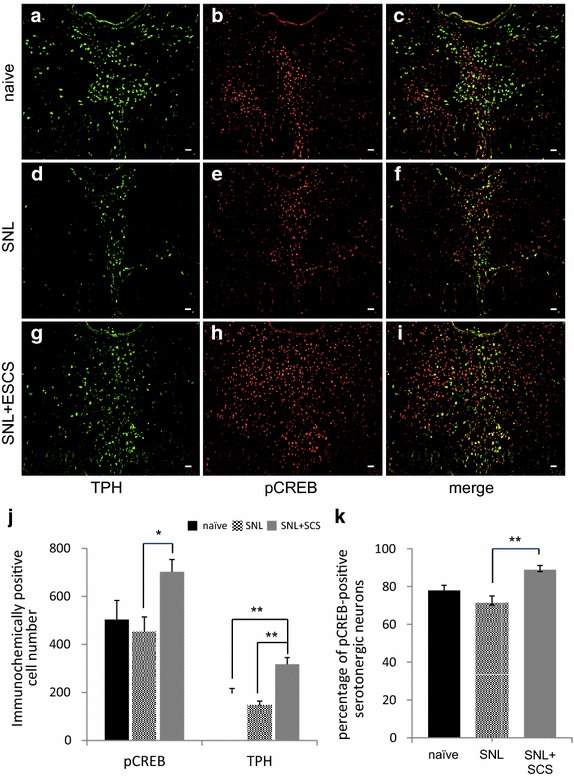
Double immunostaining of TPH and pCREB in representative DRN tissues. **a**–**c** Naïve rat; **d**–**f** SNL rat; and **g**–**i** SNL+SCS rat. The numbers of TPH-positive serotonergic neurons and pCREB-positive nuclei increased in the DRN region of the SNL+SCS rat compared with the SNL rat (*scale bar* = 20 μm). **j** Quantitative summary of pCREB-positive and TPH-positive serotonergic neurons in the DRN of the three groups. The numbers of both neuronal types in the DRN were significantly increased in SNL+SCS rats compared with SNL rats. **k** Quantitative summary of pCREB-positive serotonergic neurons among the total serotonergic neurons in the DRN. The percentage of pCREB-positive serotonergic neurons was significantly increased in SNL+SCS rats compared with SNL rats. Quantitative data were obtained from three consecutive slices of four rats per group. Data are mean ± SEM; *p < 0.05 and **p < 0.01 by the Tukey–Kramer test.

Intrathecally administered methysergide and idazoxan significantly attenuated the antinociceptive effect of SCS for at least 2 h in the ipsilateral hind paw [(baseline PWT = 15.5 ± 1.3 g; after 1-h SCS = 31.3 ± 4.3 g; 1 and 2 h after methysergide administration = 19.1 ± 1.1 and 23.4 ± 1.7 g, respectively; *F* [3, 20] = 9.33; *p* = 0.0005; Figure 1c) and (baseline PWT = 20.3 ± 2.4 g; after 1-h SCS = 30.8 ± 2.6 g; 1 and 2 h after idazoxan administration = 22.6 ± 2.6 and 22.5 ± 2.4 g, respectively; *F* [3, 24] = 3.43; *p* = 0.033; Figure 1d)]. However, they did not affect contralateral PWT [(methysergide: baseline PWT difference, *p* < 0.001; stimulated PWT difference, *p* = 0.0014; interaction of side and treatment, *p* < 0.001; n = 6) and (baseline PWT difference, *p* = 0.011; stimulated PWT difference, *p* = 0.0013; interaction of side and treatment, *p* = 0.078; n = 7)]. Furthermore, neither drug potentiated hyperalgesia in the ipsilateral or contralateral hind paws at the dose given (30 μg) (Figure 2b, c).

### SCS activated serotonergic neurons in the DRN

Spinal nerve-ligated itself did not affect the numbers of TPH-positive and pCREB-positive serotonergic neurons in the DRN. However, their numbers were significantly increased in 3-h stimulated rats compared with naïve and SNL rats [(TPH-positive serotonergic neurons: naïve, 196.1 ± 20.4; SNL, 146.6 ± 17.6; SNL + SCS, 317.9 ± 28.1; *F* [2, 31] = 17.1; *p* < 0.0001) and (pCREB-positive serotonergic neurons: naïve, 503.7 ± 79.7; SNL, 451.5 ± 63.2; SNL + SCS, 702.3 ± 51.2; *F* [2, 31] = 4.21; *p* < 0.05; Figure 2j)]. Moreover, the percentage of pCREB-positive serotonergic neurons was significantly increased in stimulated rats compared to that in the positive controls (naïve, 77.9 ± 2.7%; SNL, 71.2 ± 4.1%; SNL + SCS, 88.9 ± 2.4%; *F* [2, 24] = 8.71; *p* < 0.001 for SNL vs. SNL+SCS; Figure 2k).

### SCS did not activate noradrenergic neurons in the LC but reversed upregulated DβH expression on the ipsilateral side

**Figure 3:**
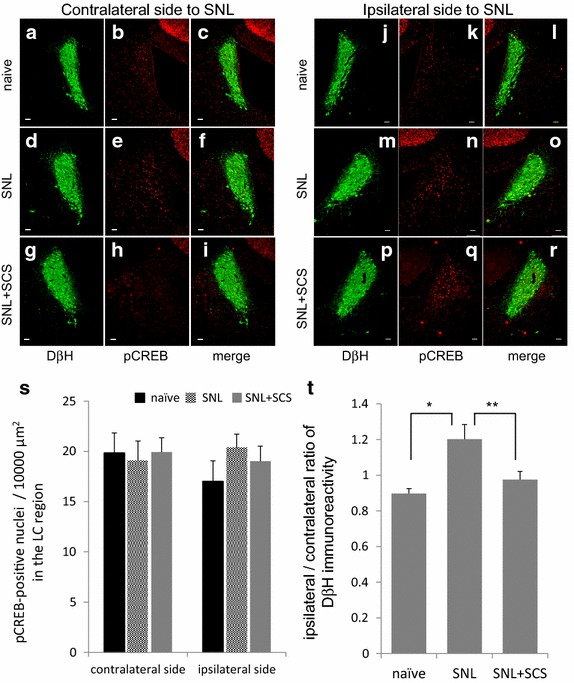
Double immunostaining of DβH and pCREB in representative bilateral LC tissues. **a**–**c** Naïve rat, contralateral side; **j**–**l** naïve rat, ipsilateral side; **d**–**f** SNL rat, contralateral side; **m**–**o** SNL rat, ipsilateral side; **g**–**i** SNL+SCS rat, contralateral side; **p**–**r** SNL+SCS rat, ipsilateral side. **s** Quantitative summary of pCREB-positive neurons in bilateral LC regions. No significant difference in the number of pCREB-positive neurons was noted among the groups. **t** Quantitative summary of the ipsilateral-to-contralateral ratio of pCREB-positive neurons and DβH immunoreactivity in bilateral LC regions. Quantitative data were obtained from three consecutive slices of four animals per group. Data are mean ± SEM; *p < 0.05 and **p < 0.01 by the Tukey–Kramer test.

The numbers of pCREB-positive nuclei (per 10,000 µm^2^) in the ipsilateral and contralateral sides of the LC were not significantly different among the groups (naïve: contralateral, 19.8 ± 2.0; ipsilateral, 17.0 ± 2.0; SNL: contralateral, 19.1 ± 2.0; ipsilateral side, 20.3 ± 1.4; SNL + SCS: contralateral, 19.9 ± 1.4; ipsilateral, 19.0 ± 1.5; *F* [1, 41] = 1.43; *p* = 0.25; Figure 3s). However, the ipsilateral-to-contralateral immunoreactivity ratio of DβH increased in the LC of SNL rats. SCS for 3 h returned the ratio to that seen in naïve rats (naïve, 0.89 ± 0.03; SNL, 1.29 ± 0.09; SNL + SCS, 0.99 ± 0.06; *p* < 0.01; Figure 3t).

### SCS did not increase the expression of TPH and DβH in the spinal dorsal horn

**Figure 4:**
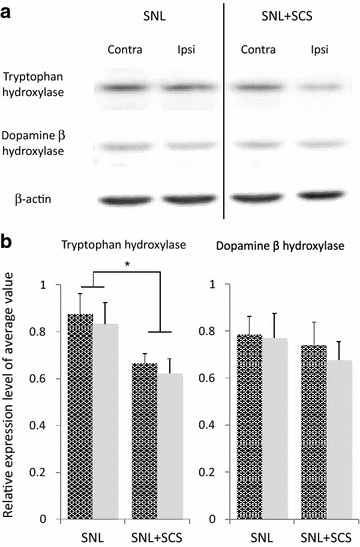
Western blotting for TPH and DβH in the spinal dorsal horn with or without SCS. **a** Representative western blot of TPH and DβH from SNL rats with or without SCS. For the SNL+SCS group, the spinal cords were harvested after 3-h SCS. **b** Summary of the densitometric analysis of western blots of TPH and DβH (n = 6 in each group). “Ipsi” and “Contra” indicate the ipsilateral and the contralateral sides in SNL rats, respectively. No significant intragroup differences were noted. Data are the ratio of each intensity to the average intensity of intermembrane control and are shown as mean ± SEM; *p < 0.05 by unpaired t test.

In all groups, no difference in enzymatic expression was noted between the ipsilateral and the contralateral sides (TPH: *p* = 0.58; DβH: *p* = 0.67), Therefore, we analyzed pooled data from the ipsilateral and contralateral sides to the nerve injury relative to the average value of intermembrane controls. The TPH expression levels in SNL rats receiving SCS were significantly lower than those in SNL rats that did not receive SCS. The DβH expression levels were unaffected by SCS. (SNL, contralateral side: 0.87 ± 0.09, ipsilateral side: 0.83 ± 0.09; SNL+SCS, contralateral side: 0.66 ± 0.04, ipsilateral side: 0.62 ± 0.06, *F* [1, 47] = 8.16; *p* = 0.007), but did not affect the DβH expression levels (SNL, contralateral side: 0.78 ± 0.08, ipsilateral side: 0.74 ± 0.10; SNL+SCS, contralateral side: 0.77 ± 0.10, ipsilateral side: 0.66 ± 0.08, *F* [1, 47] = 0.59; *p* = 0.44; Figure 4).

## Discussion

In this study, we found that SCS increased the number of and activated serotonergic neurons in the DRN. It did not activate noradrenergic neurons in the LC but restored the ipsilateral-to-contralateral ratio of DβH expression in the LC. These results evidence that the antinociceptive effect of SCS is mediated primarily by the serotonergic pathway supraspinal to the DAS and the noradrenergic pathway may have a smaller contribution, as recently suggested [29]. Moreover, the antinociceptive effect of SCS might not be mediated by local 5-HT and/or norepinephrine production, because TPH and DβH expression in the spinal dorsal horn did not increase by SCS in SNL rats.

### Supraspinal serotonergic contribution to SCS-induced antinociception

A previous study showed that peripheral nerve injury reduces 5-HT content in the ipsilateral spinal dorsal horn and SCS increases this content in responsive rats [30]. Intrathecal administration of a subanalgesic dose of 5-HT elicits an antinociceptive response in animals not responsive to SCS [5]. Moreover, the antinociceptive effect of SCS is attenuated by 5-HT_2A_ and 5-HT_4_ receptor antagonists [31]. Accordingly, our behavioral study clearly showed serotonergic involvement in the antinociceptive effect of SCS.

Electrical stimulation of brain stem nuclei suppresses spinal nociceptive reflexes and attenuates nociceptive transmission by activating the serotonergic pathway of the DAS [6]. In this study, we found that 3-h SCS increased the numbers of TPH-expressing serotonergic neurons and pCREB-positive (activated) neurons, and the percentage of pCREB-positive serotonergic neurons in the DRN of injured rats. The DRN is located adjacent to the PAG and consists of dense serotonergic neuronal clusters, which project to the NRM at the RVM [32, 33]. The NRM is known to have crucial roles in the serotonergic pathway of the DAS, because electrical stimulation of the RVM (NRM) enhances inhibitory synaptic transmission in the spinal dorsal horn [10] and induces antinociception [12]. Moreover, peripheral nerve injury attenuates the antinociceptive effect of morphine and induced neuropathic pain behavior and decreases 5-HT concentration in the NRM [34]. A recent study has shown that “off cell”-like neurons in the RVM are suppressed in neuropathic pain model rats, but SCS activates them and 5-HT-like neurons only in responsive rats [35]. By this study, we firstly showed that serotonergic neurons in the DRN (those neurons projecting into the RVM) are activated by SCS. The DRN is thought to be a major 5-HT source in the CNS [36] and connects directly to the LC, providing a potent negative input [37]. Therefore, DRN activities in a neuropathic pain state may influence both the serotonergic and the noradrenergic pathways of the DAS.

### Supraspinal noradrenergic contribution to SCS-induced antinociception

The noradrenergic pathway of the DAS has been suggested to be important for intrinsic antinociception in a neuropathic pain state and its facilitation may lead to anti-hyperalgesia, because noradrenergic neurons in the LC are activated in peripheral nerve-injured rats [13]. Furthermore, an α_2_-adrenergic agonist potentiates the antinociceptive effect of SCS [24]. By this study, we showed that the intrathecally administered α_2_-adrenergic receptor antagonist completely abolished the antinociceptive effect of SCS. In addition, we found that the immunoreactivity of DβH in the ipsilateral LC of injured rats significantly increased but was restored by 3-h SCS, although the LC neurons were not activated by SCS in SNL rats. Similarly, unilateral nerve injury does not influence the number of Fos-positive neurons in the LC if innocuous mechanical stimuli are not applied [38]. Immunohistochemical changes in the LC region in neuropathic animal models are controversial. Previous studies showed that unilateral nerve injury induces bilateral activation or excitability of LC neurons [13, 38, 39]; however, another study showed that DβH-positive neurons were increased in the LC on the ipsilateral side to sciatic nerve injury [14]. The LC receives only little input from fibers of the DRN, although noradrenergic neurons in the LC project to almost all the regions in the CNS [37]. Bilateral activation of noradrenergic neurons in the LC may be explained by projection from the DRN to both sides of the LC [37, 40]. We could not determine why the increase in DβH immunoreactivity was ipsilateral. However, the differences in the ratio of DβH expression in the LC among the three groups were relatively small and intrathecal administration of idazoxan without SCS did not alter ipsilateral PWT. The results of the behavioral experiment agree with the evidence that chronic pain does not elicit tonic activity of LC neurons and secretion of norepinephrine in the spinal cord [38, 40, 41]. Therefore, the expression change of DβH in the ipsilateral LC may only have a minor effect on the noradrenergic pathway of the DAS. However, further research is needed to elucidate the roles of LC activation in the modulation of the noradrenergic antinociceptive pathway.

Recently, Song et al. [29] have clearly shown that LC activation plays only a minor role in the antinociceptive effect of SCS, despite excitation of LC neurons in SCS-responsive neuropathic pain model rats. Our results similarly indicate that the noradrenergic pathway might play a secondary role.

### Local 5-HT and norepinephrine syntheses

The results of this study suggest that the antinociceptive effect of SCS is mediated by both serotonergic and noradrenergic mechanisms in the spinal cord, but local synthesis of 5-HT and norepinephrine might not increase after SCS in the SNL rats. A previous study revealed bilateral increase in 5-HT immunoreactivity in the superficial dorsal horn after SCS, but the SCS-induced increase in the total 5-HT content was predominantly on the ipsilateral side to nerve injury [5]. Contrarily, the results of our western blot analyses showed that the expression level of TPH decreased after SCS in the spinal dorsal horn of SNL rats. Sources of 5-HT in the spinal dorsal horn during SCS are supraspinal nuclei of serotonergic neurons, as is the RVM [35]. Moreover, 5-HT production is absent in spinal cord segments below the site of total cord transection [26]. Taken together, these results strengthened the concept that the antinociceptive effect of SCS is achieved by supraspinal facilitation of the DAS [29, 35]. However, previous studies have also shown distinct segmental effects in antinociception by SCS in neuropathic pain model rats [27, 28], suggesting that both spinal and supraspinal mechanisms are involved in SCS-induced antinociception. Our study indicated that 5-HT and norepinephrine might have smaller contributions in antinociceptive mechanisms in spinal cord segments. Segmental mechanisms of SCS antinociception are a recently proposed concept [27, 28]; further investigation is required to explain the complete mechanisms of antinociception by SCS. Controversially, both serotonergic and noradrenergic mechanisms are reported to be involved in the pain-facilitatory pathways [38, 42–44]. Differences in receptor subunits and signal transduction may account for these reported discrepancies. Molecular mechanisms involved in serotonergic and noradrenergic pain modulation need to be investigated in detail.

## Limitations

First, in this study, we used naïve animals and not sham-operated animals. Unilateral nerve injury is known to affect contralateral PWT, because nerve injury may have bilateral effects. Therefore, the contralateral limb may not be comparable to a sham-operated limb or the limb of a naïve animal. In addition, hypersensitivity in one limb may artificially produce changes in the threshold of the contralateral limb: if the animal cannot bear weight on the injured limb, it needs to put more weight on the contralateral limb. However, contralateral PWT was unchanged regardless of whether SCS significantly increased ipsilateral PWT or was not applied. Moreover, the behavioral experiment was aimed at determining serotonergic and noradrenergic contributions to the antinociceptive effect of SCS, not considering the ipsilateral-to-contralateral relationship in SCS-induced antinociception. Furthermore, sham operation may affect immunohistochemical and western blot observations because tissue damage may induce inflammation of the ipsilateral limb, which affects protein expression and glial cell activation in the spinal cord [45].

Second, we did not directly measure 5-HT and norepinephrine contents by microdialysis or enzyme-linked immunosorbent assay. Previous studies already measured these contents in the spinal dorsal horn [29, 31]. Several studies revealed that however, no report has described the expression of the synthetic enzymes of 5-HT and norepinephrine in the spinal cord of unilaterally injured animals. Our findings strengthen the concept that SCS may activate the DAS, causing antinociception in the spinal dorsal horn.

## Conclusions

SCS-induced antinociception is mediated by both the serotonergic and the noradrenergic pathways of the DAS in rats with unilateral spinal nerve injury. SCS causes histological changes in the DRN and LC, but it does not induce the expression of the synthetic enzymes of 5-HT and norepinephrine in the spinal dorsal horn of such animals. Therefore, the antinociceptive effect of SCS is mediated by activation of supraspinal centers of the DAS and not by stimulation of spinal production of 5-HT or norepinephrine.

## Methods

### Animals

Male Sprague–Dawley rats weighing 350–400 g were used for the experiments. The animals were housed under a 12-h light/dark cycle in controlled temperature and humidity conditions and had free access to food and water. All experiments conformed to international guidelines on ethical use of animals [46], and all efforts were made to minimize pain and discomfort. Animal housing and surgical procedures were approved by the Institutional Animal Care and Use Committee of Yokohama City University Graduate School of Medicine (Approval No. F10-112). At the end of the experiments, all the animals were euthanized by an overdose of pentobarbital.

### SNL and implantation procedures

The left L5 spinal nerve was ligated as previously described [47] with some modifications. In brief, animals were anesthetized with isoflurane (1.5–2%) in oxygen, and the left L6 transverse process was removed. The left L5 spinal nerve was identified and tightly ligated using a 4-0 silk suture. The wound was then closed.

One week later, electrodes for SCS and intrathecal catheters were implanted as previously described [48]. In brief, animals anesthetized with isoflurane were placed in the supine position. After the back hair was clipped, a midline skin incision of 2–3 cm was made in the lumbar area (L2–5) and the vertebral muscles were bluntly dissected. Under binocular magnification, the L3 spinal process was removed, the L4 vertebra was retracted to expose the L2–3 and L3–4 intervertebral spaces, and the associated intervertebral ligaments were carefully cut. A silver electrode was inserted into the epidural space 2–2.5 cm cephalad from the L2–3 intervertebral space such that its tip was located in the T11–13 area. Another silver electrode was inserted 1 cm caudally from the L3–4 intervertebral space. These epidural electrodes (0.2 mm in diameter) were fabricated into ball-shaped tips and insulated by tubular polyethylene tubing (0.61 mm in outer diameter; PE-10, Becton, Dickinson and Company, Franklin Lakes. NJ).

At the same time, an intrathecal catheter (PE-10) was inserted in the same intervertebral space where the silver electrodes were inserted into the epidural space. The catheter was advanced 15 mm cephalad into the lumbar subarachnoid space so that its tip was located near the lumbar enlargement [49]. The electrodes and intrathecal catheter were then tunneled subcutaneously and externalized in the neck region. The volume of dead space of the intrathecal catheter was 15 μL.

Muscles, fascia, and skin were closed with 3-0 silk sutures in layers. The animals were typically allowed to recover for a minimum of 5 days before testing. Those showing signs of motor weakness or paresis after recovery from anesthesia were euthanized. By histopathological analysis of a harvested spinal cord, we confirmed that the electrode tips were placed on the dorsal spinal surface at the T10–11 level and left side of the same surface at the L4–5 level.

### Behavioral analysis

The behavioral assessments were performed under standardized conditions in a separate quiet room. On each testing day, rats were brought into the quiet room 1 h before the test session to allow them to habituate to the environment. Mechanical hypersensitivity was assessed with a Dynamic Plantar Aesthesiometer (Ugo Basile, Comerio, Italy), which is an automated von Frey device [50–52].

To measure hind-paw mechanical thresholds, animals were placed in plastic cages with a wire mesh floor and allowed to acclimate for 15 min before each test session. A paw-flick response was elicited by applying an increasing force using a plastic filament (0.5 mm in diameter) on the center of the plantar surface of both hind paws. The force applied was initially below the detection threshold, then increased from 1 to 50 g in 1 g increments over 20 s, and finally held at 50 g for 10 s. The rate of increase was 2.5 g/s. PWT was defined as the force applied to elicit reflex removal of the hind paw. The mean of five measurements at 1-min intervals was calculated to determine PWT of each side.

### Spinal cord stimulation (SCS)

After the baseline PWT measurement, the implanted electrodes were connected to an electrical stimulator (SEN-3301, Nihon Kohden, Tokyo, Japan) via an isolator (SS-201J, Nihon Kohden) with wire leads, allowing the animals to move freely in the cage. Monopolar electrical stimuli with a frequency of 50 Hz and pulse width of 0.2 ms were applied. The amplitude was individually determined as 70–80% of the intensity required to produce slight twitching in the lower trunk muscles or leg stretching (i.e., motor threshold), as reported in previous studies [24, 48]. We defined a response as increased PWT (>130% of the initial value) on the ipsilateral side after 1-h SCS.

### Intrathecal drug administration

We used rats showing attenuation of mechanical hypersensitivity after 1-h SCS for the subsequent experiments. After obtaining the 1-h PWT measurement, we administered 30 μg of methysergide maleate (5-HT_1_- and 5-HT_2_-receptor antagonist), idazoxan hydrochloride (α_2_-adrenergic receptor antagonist), or saline via the intrathecal catheter (total volume = 10 μL) using a Hamilton syringe, followed by 10 μL of saline to rinse the catheter. PWT was remeasured at 1 and 2 h after drug administration (Figure 1). Unstimulated SNL animals served as positive controls. The drugs were dissolved in saline and kept at −80°C before use. Their doses were determined from a previous report [53].

### Immunohistochemistry

Animals showing increased PWT after 3-h SCS, unstimulated SNL animals, and naïve rats (negative controls) (n = 4 rats per group) were deeply anesthetized with sodium pentobarbital (50 mg/kg) and perfused intracardially with heparinized phosphate-buffered saline (PBS), followed by 4% cold buffered paraformaldehyde with 2% saturated picric acid solution. The brains were rapidly removed, fixed in the same fixative solution at 4°C overnight, and cryoprotected in 30% sucrose solution at 4°C until they completely sank. The medullae, containing the DRN (bregma 7.30–7.80 mm [54]) and LC (bregma 9.68–10.04 mm [54]), were sliced in 20-µm sections and mounted on Frontier-coated glass slides (Matsunami Glass, Osaka, Japan). Then, sections were incubated overnight in a mixture of primary antibodies: sheep anti-TPH (1:400; Sigma-Aldrich, St. Louis, MO) and rabbit anti-serine 133 pCREB (1:1,000; Upstate, EMD Millipore, Billerica, MA) for DRN-containing slices or mouse anti-DβH (1:400; EMD Millipore) and anti-pCREB (1:1,000; Upstate, EMD Millipore, Billerica, MA) for LC-containing slices. The following secondary antibodies were used: donkey anti-sheep immunoglobulin G coupled to fluorescein isothiocyanate (FITC) for TPH (1:400), donkey anti-mouse coupled to DyLight 488 for DβH (1:400), and donkey anti-rabbit coupled to DyLight 549 for pCREB (1:400) (Jackson ImmunoResearch, West Grove, PA).

Immunofluorescent reactions were observed under an all-in-one epifluorescence microscope (Biorevo BZ-9000, Keyence Corporation, Osaka, Japan) equipped with excitation filters for tetramethylrhodamine (TRITC) and FITC fluorescence wavelengths. Images of DRN- and LC-containing slices, identified by TPH- and DβH-positive neuronal clusters, respectively, were captured using a digital camera containing a charge-coupled device and connected to the epifluorescence microscope system. Two to three sections were chosen from within 80 µm of the rostral-to-caudal extents of the DRN and LC per rat for analysis with ImageJ software (National Institutes of Health, Bethesda, MA) after binarization. TRITC-stained ovoid particles were considered as pCREB-positive nuclei. All FITC-stained cell bodies, with or without stained nuclei, were considered as TPH-positive neurons. The percentage of pCREB-positive serotonergic neurons was calculated from the total number of TPH-positive neurons counted visually. Further, the average fluorescence intensity (arbitrary units from 0 to 255 per pixel) of DβH-positive neurons and number of pCREB-positive nuclei were quantified in the chosen LC sections. The ipsilateral-to-contralateral immunoreactivity ratio of DβH was then calculated. The immunohistochemical analyses were performed by two researchers (KS and AY) in a blinded manner.

### Western blot analysis

Animals showing increased PWT after 3-h SCS (n = 6), unstimulated SNL animals (n = 6) were deeply anesthetized with sodium pentobarbital (50 mg/kg) and perfused intracardially with heparinized cold PBS. The L4–5 segment of the spinal cord was rapidly removed and divided into quarters (left dorsal, left ventral, right dorsal, and right ventral). The dorsal quarters were immediately but separately frozen in liquid nitrogen and stored at −80°C until use. Frozen samples were homogenized with ice-cold solubilizing buffer (10-mM hydroxyethyl piperazineethanesulfonic acid, 1.0-mM ethylenediaminetetraacetic acid, 2.0-mM ethylene glycol tetraacetic acid, 0.5-mM dithiothreitol, 0.1-mMphenylmethanesulfonyl fluoride, 10-mg/L leupeptin, and 100-nM microcystin) and sonicated and boiled at 97°C for 5 min.

Protein concentration was determined by using a BCA reagent assay kit (Pierce, Thermo Fisher Scientific, Rockford, IL). Protein samples (10 μg/well for the dorsal quarters, 5 μg/well for each brain) were subjected to 8% polyacrylamide gel electrophoresis in the presence of sodium dodecyl sulfate and transferred electrophoretically to polyvinylidene difluoride membranes. The membranes were incubated in a blocking solution, 5% skim milk dissolved in Tris-buffered saline containing 0.1% Tween-20 (pH 7.4), for 1 h at room temperature. They were then incubated with anti-TPH antibody (1:2,000; Sigma-Aldrich), anti-DβH antibody (1:1,000; Cell Signaling Technology, Danvers, MA), and anti-β-actin monoclonal antibody (1:20,000, Sigma-Aldrich) overnight in the blocking solution at 4°C. Subsequently, they were washed with Tris-buffered saline containing 0.1% Tween-20 and incubated in donkey anti-rabbit and sheep anti-mouse horseradish peroxidase (both 1:20,000; GE Healthcare, Piscataway, NJ) in the blocking solution for 1 h at room temperature. Immunodetection was performed with ECL Advance reagents (GE Healthcare) according to the manufacturer’s instructions and quantified by densitometry using LAS3000 and Multi Gauge software (Fujifilm, Tokyo, Japan). For each band, blank values were first subtracted. Variations between gels were controlled by expressing the results as a ratio to the intermembrane control. Data were calculated as ratios of each intensity to the average value of intermembrane control.

### Statistical analysis

Behavioral data are shown as means (g) ± standard error of the mean (SEM). Immunohistochemical data are shown as mean ± SEM of actual cell counts in the DRN or ipsilateral-to-contralateral ratio of DβH optical density in the LC. Western blot data are shown as mean ± SEM of relative optical density. Two-way repeated-measures analysis of variance (ANOVA) was used to analyze the effects of SCS and the drugs on PWT in each group; then, one-way repeated-measures ANOVA followed by the Dunnett test were used for intergroup behavioral comparisons. Immunohistochemical data were compared among the groups by one-way ANOVA followed by the Tukey–Kramer test. Western blot data between SNL and SNL+SCS groups were compared by two-way repeated-measures ANOVA followed by unpaired t tests. Values of *p* less than 0.05 were considered significant. The data were analyzed using the XLSTAT Microsoft Excel macro (ver. 2015. 2.01.16567, Addinsoft, New York, NY).

## Abbreviations

ANOVA: Analysis of variance
DAS: descending antinociceptive system
DβH: dopamine β-hydroxylase
DRN: dorsal raphe nucleus
5-HT: 5-hydroxytryptamine
FITC: fluorescein isothiocyanate
GABA: gamma-aminobutyric acid
LC: locus coeruleus
NRM: nucleus raphe magnus
PAG: periaqueductal gray matter
PBS: phosphate-buffered saline
pCREB: phosphorylated cyclic AMP response element binding protein
RVM: rostral ventromedial medulla
SCS: spinal cord stimulation
SNL: spinal nerve ligation
TPH: tryptophan hydroxylase
TRITC: tetramethylrhodamine

## References

[1] Bardin L, Bardin M, Lavarenne J, Eschalier A (1997). Effect of intrathecal serotonin on nociception in rats: influence of the pain test used. Exp Brain Res.

[2] Bardin L, Jourdan D, Alloui A, Lavarenne J, Eschalier A (1997). Differential influence of two serotonin 5-HT3 receptor antagonists on spinal serotonin-induced analgesia in rats. Brain Res.

[3] Bardin L, Lavarenne J, Eschalier A (2000). Serotonin receptor subtypes involved in the spinal antinociceptive effect of 5-HT in rats. Pain.

[4] Bardin L, Schmidt J, Alloui A, Eschalier A (2000). Effect of intrathecal administration of serotonin in chronic pain models in rats. Eur J Pharmacol.

[5] Song Z, Ultenius C, Meyerson BA, Linderoth B (2009). Pain relief by spinal cord stimulation involves serotonergic mechanisms: an experimental study in a rat model of mononeuropathy. Pain.

[6] Liu MY, Su CF, Lin MT (1988). The antinociceptive role of a bulbospinal serotonergic pathway in the rat brain. Pain.

[7] Sluka KA, Lisi TL, Westlund KN (2006). Increased release of serotonin in the spinal cord during low, but not high, frequency transcutaneous electric nerve stimulation in rats with joint inflammation. Arch Phys Med Rehabil.

[8] El-Khoury C, Hawwa N, Baliki M, Atweh SF, Jabbur SJ, Saade NE (2002). Attenuation of neuropathic pain by segmental and supraspinal activation of the dorsal column system in awake rats. Neuroscience.

[9] Saade NE, Al Amin H, Chalouhi S, Baki SA, Jabbur SJ, Atweh SF (2006). Spinal pathways involved in supraspinal modulation of neuropathic manifestations in rats. Pain.

[10] Kato G, Yasaka T, Katafuchi T, Furue H, Mizuno M, Iwamoto Y (2006). Direct GABAergic and glycinergic inhibition of the substantia gelatinosa from the rostral ventromedial medulla revealed by in vivo patch-clamp analysis in rats. J Neurosci.

[11] Vanegas H, Barbaro NM, Fields HL (1984). Midbrain stimulation inhibits tail-flick only at currents sufficient to excite rostral medullary neurons. Brain Res.

[12] Sorkin LS, McAdoo DJ, Willis WD (1993). Raphe magnus stimulation-induced antinociception in the cat is associated with release of amino acids as well as serotonin in the lumbar dorsal horn. Brain Res.

[13] Hayashida K, Obata H, Nakajima K, Eisenach JC (2008). Gabapentin acts within the locus coeruleus to alleviate neuropathic pain. Anesthesiology.

[14] Ma W, Eisenach JC (2003). Chronic constriction injury of sciatic nerve induces the up-regulation of descending inhibitory noradrenergic innervation to the lumbar dorsal horn of mice. Brain Res.

[15] Wallace M, Yaksh TL (2000). Long-term spinal analgesic delivery: a review of the preclinical and clinical literature. Reg Anesth Pain Med.

[16] Uhle EI, Becker R, Gatscher S, Bertalanffy H (2000). Continuous intrathecal clonidine administration for the treatment of neuropathic pain. Stereotact Funct Neurosurg.

[17] Siddall PJ, Molloy AR, Walker S, Mather LE, Rutkowski SB, Cousins MJ (2000). The efficacy of intrathecal morphine and clonidine in the treatment of pain after spinal cord injury. Anesth Analg.

[18] Kawamata T, Omote K, Kawamata M, Iwasaki H, Namiki A (1997). Antinociceptive interaction of intrathecal alpha2-adrenergic agonists, tizanidine and clonidine, with lidocaine in rats. Anesthesiology.

[19] Kawamata T, Omote K, Yamamoto H, Toriyabe M, Wada K, Namiki A (2003). Antihyperalgesic and side effects of intrathecal clonidine and tizanidine in a rat model of neuropathic pain. Anesthesiology.

[20] Pan HL, Chen SR, Eisenach JC (1998). Role of spinal NO in antiallodynic effect of intrathecal clonidine in neuropathic rats. Anesthesiology.

[21] Field MJ, McCleary S, Hughes J, Singh L (1999). Gabapentin and pregabalin, but not morphine and amitriptyline, block both static and dynamic components of mechanical allodynia induced by streptozocin in the rat. Pain.

[22] Hayashida K, DeGoes S, Curry R, Eisenach JC (2007). Gabapentin activates spinal noradrenergic activity in rats and humans and reduces hypersensitivity after surgery. Anesthesiology.

[23] Kumar N, Laferriere A, Yu JS, Leavitt A, Coderre TJ (2010). Evidence that pregabalin reduces neuropathic pain by inhibiting the spinal release of glutamate. J Neurochem.

[24] Schechtmann G, Wallin J, Meyerson BA, Linderoth B (2004). Intrathecal clonidine potentiates suppression of tactile hypersensitivity by spinal cord stimulation in a model of neuropathy. Anesth Analg.

[25] Cui M, Feng Y, McAdoo DJ, Willis WD (1999). Periaqueductal gray stimulation-induced inhibition of nociceptive dorsal horn neurons in rats is associated with the release of norepinephrine, serotonin, and amino acids. J Pharmacol Exp Ther.

[26] Li Y, Li L, Stephens MJ, Zenner D, Murray KC, Winship IR (2014). Synthesis, transport, and metabolism of serotonin formed from exogenously applied 5-HTP after spinal cord injury in rats. J Neurophysiol.

[27] Barchini J, Tchachaghian S, Shamaa F, Jabbur SJ, Meyerson BA, Song Z (2012). Spinal segmental and supraspinal mechanisms underlying the pain-relieving effects of spinal cord stimulation: an experimental study in a rat model of neuropathy. Neuroscience.

[28] Smits H, van Kleef M, Joosten EA (2012). Spinal cord stimulation of dorsal columns in a rat model of neuropathic pain: evidence for a segmental spinal mechanism of pain relief. Pain.

[29] Song Z, Ansah OB, Meyerson BA, Pertovaara A, Linderoth B (2013). Exploration of supraspinal mechanisms in effects of spinal cord stimulation: role of the locus coeruleus. Neuroscience.

[30] Liu FY, Qu XX, Ding X, Cai J, Jiang H, Wan Y (2010). Decrease in the descending inhibitory 5-HT system in rats with spinal nerve ligation. Brain Res.

[31] Song Z, Meyerson BA, Linderoth B (2011). Spinal 5-HT receptors that contribute to the pain-relieving effects of spinal cord stimulation in a rat model of neuropathy. Pain.

[32] Kwiat GC, Basbaum AI (1990). Organization of tyrosine hydroxylase- and serotonin-immunoreactive brainstem neurons with axon collaterals to the periaqueductal gray and the spinal cord in the rat. Brain Res.

[33] Cho HJ, Basbaum AI (1991). GABAergic circuitry in the rostral ventral medulla of the rat and its relationship to descending antinociceptive controls. J Comp Neurol.

[34] Sounvoravong S, Nakashima MN, Wada M, Nakashima K (2004). Decrease in serotonin concentration in raphe magnus nucleus and attenuation of morphine analgesia in two mice models of neuropathic pain. Eur J Pharmacol.

[35] Song Z, Ansah OB, Meyerson BA, Pertovaara A, Linderoth B (2013). The rostroventromedial medulla is engaged in the effects of spinal cord stimulation in a rodent model of neuropathic pain. Neuroscience.

[36] Dahlstrom A, Fuxe K (1964). Localization of monoamines in the lower brain stem. Experientia.

[37] Kim MA, Lee HS, Lee BY, Waterhouse BD (2004). Reciprocal connections between subdivisions of the dorsal raphe and the nuclear core of the locus coeruleus in the rat. Brain Res.

[38] Brightwell JJ, Taylor BK (2009). Noradrenergic neurons in the locus coeruleus contribute to neuropathic pain. Neuroscience.

[39] Takasu K, Ono H, Tanabe M (2008). Gabapentin produces PKA-dependent pre-synaptic inhibition of GABAergic synaptic transmission in LC neurons following partial nerve injury in mice. J Neurochem.

[40] Alba-Delgado C, Borges G, Sanchez-Blazquez P, Ortega JE, Horrillo I, Mico JA (2012). The function of alpha-2-adrenoceptors in the rat locus coeruleus is preserved in the chronic constriction injury model of neuropathic pain. Psychopharmacology.

[41] Viisanen H, Pertovaara A (2007). Influence of peripheral nerve injury on response properties of locus coeruleus neurons and coeruleospinal antinociception in the rat. Neuroscience.

[42] Rahman W, Suzuki R, Webber M, Hunt SP, Dickenson AH (2006). Depletion of endogenous spinal 5-HT attenuates the behavioural hypersensitivity to mechanical and cooling stimuli induced by spinal nerve ligation. Pain.

[43] Wei F, Dubner R, Zou S, Ren K, Bai G, Wei D (2010). Molecular depletion of descending serotonin unmasks its novel facilitatory role in the development of persistent pain. J Neurosci.

[44] Calejesan AA, Ch’ang MH, Zhuo M (1998). Spinal serotonergic receptors mediate facilitation of a nociceptive reflex by subcutaneous formalin injection into the hindpaw in rats. Brain Res.

[45] Blackbeard J, O’Dea KP, Wallace VC, Segerdahl A, Pheby T, Takata M (2007). Quantification of the rat spinal microglial response to peripheral nerve injury as revealed by immunohistochemical image analysis and flow cytometry. J Neurosci Methods.

[46] Zimmermann M (1983). Ethical guidelines for investigations of experimental pain in conscious animals. Pain.

[47] Kim SH, Chung JM (1992). An experimental model for peripheral neuropathy produced by segmental spinal nerve ligation in the rat. Pain.

[48] Kakinohana M, Harada H, Mishima Y, Kano T, Sugahara K (2005). Neuroprotective effect of epidural electrical stimulation against ischemic spinal cord injury in rats: electrical preconditioning. Anesthesiology.

[49] Pogatzki EM, Zahn PK, Brennan TJ (2000). Lumbar catheterization of the subarachnoid space with a 32-gauge polyurethane catheter in the rat. Eur J Pain.

[50] Kalmar B, Greensmith L, Malcangio M, McMahon SB, Csermely P, Burnstock G (2003). The effect of treatment with BRX-220, a co-inducer of heat shock proteins, on sensory fibers of the rat following peripheral nerve injury. Exp Neurol.

[51] Lever I, Cunningham J, Grist J, Yip PK, Malcangio M (2003). Release of BDNF and GABA in the dorsal horn of neuropathic rats. Eur J Eurosci.

[52] Obata K, Katsura H, Sakurai J, Kobayashi K, Yamanaka H, Dai Y (2006). Suppression of the p75 neurotrophin receptor in uninjured sensory neurons reduces neuropathic pain after nerve injury. J Neurosci.

[53] Obata H, Saito S, Koizuka S, Nishikawa K, Goto F (2005). The monoamine-mediated antiallodynic effects of intrathecally administered milnacipran, a serotonin noradrenaline reuptake inhibitor, in a rat model of neuropathic pain. Anesth Analg.

[54] Paxinos G, Watson C (2005). The rat brain in stereotaxic coordinates.

